# Factors influencing COVID-19 knowledge-gap: a cross-sectional study in China

**DOI:** 10.1186/s12889-021-11856-9

**Published:** 2021-10-09

**Authors:** Han Wang, Lina Li, Jing Wu, Hao Gao

**Affiliations:** 1 School of Journalism and Communication, Jinan University, No.601, West Huangpu Avenue Guangzhou, Guangdong, 510632 People’s Republic of China; 2 Film-Television and Communication College, Shanghai Normal University, Guilin Road, Xuhui District, Shanghai, 200233 People’s Republic of China; 3 Faculty of Social Sciences, University of Ljubljana, Kardeljeva ploščad, SI-1000 Ljubljana, Slovenia; 4 School of Journalism and Communication, Nanjing Normal University, Ninghai Road, Gulou District, Nanjing, 210097 People’s Republic of China

**Keywords:** The COVID-19 knowledge gap, Internet usage, Perceived salience of information

## Abstract

**Background:**

In the face of a sudden outbreak of COVID-19, it is essential to promote health communication, especially to reduce communication inequality. The paper targeted China to investigate whether social structural factors (education level and urban-rural differences) lead to the knowledge gap of COVID-19. Also, this paper examined whether media use, interpersonal communication, public communication, and perceived salience of information can influence the knowledge gap of COVID-19. Furthermore, this paper explored the strategies to promote communication equality.

**Methods:**

An online survey on COVID-19 knowledge and its influencing factors was conducted in February 2020, with a valid sample of 981 participants. The dependent variable was the total score of knowledge related to COVID-19. In addition to demographic variables such as education level and residence, the main explanatory variables include four independent variables: the use of different media (print media, radio, television, Internet), interpersonal communication, public communication, and perceived salience of information. This paper utilized descriptive statistics, correlation analysis, and hierarchical multiple regression analysis for data processing.

**Results:**

Descriptive statistics indicated that the Internet was the most frequent source of information for participants to obtain COVID-19 knowledge (M = 6.28, SD = 1.022). Bi-variate analysis and regression analysis presented that education level, Internet media use, and perceived salience of information predicted the difference in knowledge level. Hierarchical multiple regression showed that Internet media use significantly predicted differences in the level of knowledge related to COVID-19 among groups with different education levels.

**Conclusions:**

This study found a COVID-19 knowledge gap among the Chinese public, especially the digital knowledge gap. Education level, perceived salience of information, and internet media use can significantly predict the difference in COVID-19 knowledge level. In contrast, the use of traditional media such as newspaper, radio, and television, public communication, and interpersonal communication did not improve knowledge level. Internet media use and education level have an interactive effect on the formation of a COVID-19 knowledge gap. That is, online media use will expand the COVID-19 knowledge gap between groups with different education levels.

## Background

A new coronavirus was isolated from airway epithelial cells of patients with unexplained pneumonia in Wuhan, China, in December 2009, after which the WHO named it COVID-19(Coronavirus Disease, 2019). The discovery of COVID-19 and its subsequent widespread made it a severe public health emergency. In previous studies on AIDS [1], influenza [2], and other infectious diseases, scholars generally believed that people with a complete understanding of infectious diseases could accurately assess the threat of viruses and take active preventive actions. It is proved that mass media effectively changed individual health behaviors [3–7] and improved cognition of disease symptoms and signs [8]. In the face of unknown new viruses and diseases, people need to acquire relevant knowledge to deal with them. Various media channels have become the main way for people to learn health knowledge [9]. Is there a knowledge gap in the accumulation of COVID-19 knowledge?

Techenor put forward the “knowledge gap” hypothesis in the 1970s, which argued that the dissemination of media information would increase the knowledge gap between people of different economic status (class). People with higher education levels are more capable of acquiring new information than those with lower education levels. With the increase of media information over time, people with higher education levels will get more helpful information, extending the “knowledge gap” between the two classes [10].

In the era of the “knowledge gap,” mass media was the essential channel for obtaining information, no matter for people of low or high economic status [10]. The rise of the Internet changed the way of personal information acquisition and knowledge construction, which improved access to health information. People preferred searching and sharing information through the Internet [11]. Therefore, the media influence mechanism to form the “knowledge gap” is different from the mass media era. During the COVID-19, Internet media, with its advantages in timeliness, convenience, and user scale, has become the most important way to release and disseminate relevant information [12]. Besides, traditional media such as radio, television, newspapers and magazines, and public communication are also significant channels for spreading information about the COVID-19 [13, 14]. Under the influence of multiple media channels, COVID-19 has become the most concerned issue among the public. People also track, communicate, and discuss this issue through interpersonal communication [15].

Hence, it is of practical significance to re-examine the knowledge gap and Health Communication in COVID-19. This study will focus on the following questions: Is there a knowledge gap among the Chinese public regarding the COVID-19? If so, what are the influencing factors? How to eliminate inequalities in health communication?

The “knowledge gap” hypothesis suggested that the difference in socioeconomic status (SES) would lead to unequal access to education, leading to unequal access to knowledge in the face of mass information flow [10]. This study took the education level as the primary basis for predicting the difference in knowledge levels. Then, it proposed a hypothesis that the higher educated group has a higher level of COVID-19 knowledge than the less educated group [Hypothesis 1]. Specifically, it is essential to explore the role that differences in access to health information play in forming the health knowledge gap in health communication.

Therefore, this study will explore whether information sources such as media use, public communication, and interpersonal communication can significantly predict knowledge level gaps in the context of COVID-19. From the perspective of media use, among many media forms, Internet media is the way people rely more on upon to obtain information [16]. Therefore, this study put forward another hypothesis that Internet media can better predict the gap in knowledge level [Hypothesis 2].

In addition, Ettema and Kline (1977) believed that the rise of the knowledge gap is not due to the educational differences but the difference in the perceived salience of information. Perceived salience of information refers to people’s “beliefs about the usefulness of the information in various channels” [17]. If information is perceived to be helpful by social system members, then the education-based knowledge gap is less likely to appear [18]. Empirical studies have found that inequality in knowledge based on educational deficiencies can be improved by shifts in usefulness beliefs, such as the relevance or interest of information to individuals [19]. Therefore, it can be inferred that the less educated group can obtain more knowledge by improving information salience to narrow the knowledge gap based on educational attainment differences. Based on the perceived salience of information, as an individual information motive, this study explored whether groups with higher perceived salience of information have a higher knowledge level of COVID-19 than groups with lower perceived salience of information. So, this paper hypothesized that perceived salience of information can predict and change the knowledge gap [Hypothesis 3]. This study explored the questions and examined the hypotheses above by surveying the Chinese public.

## Methods

### Study design and participants

The data in this study were derived from an online survey on COVID-19 knowledge and its influencing factors conducted by the School of Journalism and Communication at Nanjing Normal University in February 2020. The survey was conducted on Wenjuanxing (https://www.wjx.cn/), one of China’s most professional online questionnaire platforms, calling for 1023 participants with the interpersonal snowball method. The final valid samples were 981 after eliminating invalid samples, with an effective rate of 95%, including people from all provinces in mainland China and Hong Kong. The youngest participant in the sample was over 16 years old. According to the Kendall sample estimation method for multivariate analysis, the minimum sample size was required to be ten times the number of the scale items [20]. This survey involved 33 scale items, and the sample size reached the standard. Prior to accessing the survey, participants read an informed consent statement that described that participation was voluntary and that they could stop at any time. By clicking on a “next” button, participants were informed that they were providing consent to complete the survey.

### Variable selection

#### Outcome variable

The dependent variable in this study was the level of COVID-19 knowledge. Knowledge as a variable is often measured in the knowledge gap studies by true-false questions, but the reliability is not scientific [21]. Besides, the traditional reliability and validity measurements are generally used for scale data. The questionnaire in this study drew on previous research on the knowledge gap of influenza, HIV, and other diseases [2, 10] and the official knowledge of COVID-19 characteristics, mortality, and prevention in China as of the end of February 2020 [22]. The final measurement scale of this study included 18 questions in four parts. The most prominent problem in measuring the level of knowledge is the confusion of knowledge and belief [21, 23], in which individuals might know the facts but do not believe them. Zimet suggested setting up statements beginning with “Most experts believed that … ” to avoid confusing knowledge with belief [21]. As a result, this study’s measurement items of knowledge about COVID-19 began with “Most experts believed that...”. Each question was set to three answer items with ‘correct,’ ‘wrong,’ and ‘do not know,’ with one mark for a correct answer against the knowledge and 0 marks for the remaining results. The sum for each question was the total score for COVID-19 knowledge (Range 0–18, M = 12.98, SD = 3.34).

#### Explanatory variables

##### Education level

This study measured measure socioeconomic status (SES) with educational attainment. The question in the questionnaire was: “Your highest educational background: 1. Middle school and below; 2. High school/technical secondary school; 3. Junior college; 4. Bachelor’s degree; 5. Master’s degree and above.”

##### Media use related to COVID-19

This variable refers to individuals’ intentional or unintentional use of different media (print, radio, television, Internet) to obtain information related to COVID-19 from 31st December 2019 (when Wuhan Health Commission announced unknown pneumonia) to 29th February. A seven-level scale (Cronbach’s α =0.71) consisting of four items was used, “In the past two months, how have you often used the following media to obtain information related to COVID-19?” Respondents were asked to choose from “1. Never” to “7. Always”.

##### The interpersonal communication related to COVID-19

The interpersonal communication variable was measured by a mature seven-level scale [24] with two items (Cronbach’s α =0.65): 1. How often do you participate in discussions related to knowledge/awareness about the COVID-19? 2. How often do you initiate discussions with others about COVID-19? (Choose one from “1. Never” to “7. Always”). The interpersonal communication score was the average of the two items (M = 4.85, SD = 1.33).

##### Public communication related to COVID-19

Public communication refers to the process of transmitting information and exchanging opinions with the public through various means by the government, enterprises, and other organizations [25]. This variable was measured by a seven-level scale with six items: 1. Cuncunxiang (Extending Radio Broadcasting Coverage to Every Village Project) radio; 2. Car radio; 3. Door-to-door visits by volunteers/community workers; 4. Telephone notification from volunteers/community workers; 5. SMS notification from relevant departments; 6. Posters/documents posted to publicize Covid-19 prevention knowledge. Respondents were asked to choose from “1. Never” to “7. Always.”, and the scale had good internal consistency (Cronbach’s α =0.83, M = 4.20, SD = 1.30).

##### Perceived salience of information

The three questions about the perceived salience of information were set based on relevant literature [17], including “1. I believe that the COVID-19 information transmitted through different channels is closely related to me; 2. I believe that the COVID-19 information transmitted through different channels is closely related to my friends, family, and community residents; 3. I feel that the information about COVID-19 transmitted through different channels is helpful for my current situation.” Respondents were asked to choose one from “1. Strongly disagree” to “7. Strongly agree.”, and the scale had good internal consistency (Cronbach’s α = 0.88, M = 6.13, SD = 1.12).

### Data analysis

A total of 981 valid samples were used for data analysis. We checked outliers and multicollinearity before the analysis, re-coded and standardized variables to fit the study design. SPSS V.26 was used for descriptive statistics, bivariate correlation analysis, and multi-level regression analysis. Descriptive analysis used percentages to describe the variables. Bivariate correlation analysis explored whether the dependent variables have significant positive correlations with the knowledge level. Finally, three models were used to constitute a hierarchical regression analysis to examine whether variables positively correlated with the knowledge level (R > 0.1, *P* < 0.05) could significantly predict the gap of COVID-19 knowledge level in the total sample. Also, the hierarchical regression analysis explored whether the affecting variables expand or narrow the knowledge gap between groups with different educational levels.

## Results

### Descriptive statistical analysis

The demographic characteristics of 981 valid samples are shown in Table 1, in which the education level covers five different levels from junior high school or below to postgraduate. The levels of COVID-19 knowledge are shown in Table 2 that the correct average rate of knowledge score was 73.6%. In terms of sources of knowledge acquisition (Table 3), the Internet media was the most frequent information source (M = 6.28, SD = 1.022), followed by interpersonal communication (M = 4.854, SD = 1.333), radio and television (M = 4.446, SD = 1.463), and public communication (M = 4.198, SD = 1.304). Print media (M = 3.118, SD = 1.827) had the lowest frequency. Overall, the perceived salience of information was of high significance (M = 6.133, SD = 1.122).

**Table 1:** Sociodemographic information of the participants (*N* = 981)

Variable	n(%)or Mean ± SD
**Sex**	
Male	438 (44.65)
Female	543 (55.35)
**Age (year)**	28.063 ± 9.451
**Education level**	
Middle school and below	71 (7.24)
High school/technical secondary school	81 (8.26)
Junior college	150 (15.29)
Bachelor’s degree	490 (49.95)
Master’s degree and above	189 (19.27)
**Occupation**	
Student	349 (35.58)
Worker	22 (2.24)
Farmer	3 (0.31)
Self-employed	50 (5.10)
Employee of enterprise or institution	442 (45.06)
Retired	5 (0.51)
Unemployed	25 (2.55)
Other	85 (8.66)
**Place of residence**	
City	816 (83.18)
Countryside or town	165 (16.82)

**Table 2:** Knowledge level of respondents regarding COVID-19 (*N* = 981)

Items	Accuracy n(%)
**Recognition of COVID-19**	
1.COVID-19 is an acute respiratory infection characterized by fever, dry cough, and fatigue.	857 (87.4)
2.The genetic characteristics of COVID-19 are not significantly different from those of SARS (Severe Acute Respiratory Syndrome).	621 (63.3)
3.COVID-19 was not sensitive to heat.	676 (68.9)
4.All infected cases present with symptoms of fever.	621 (63.3)
5.People generally susceptible to COVID −19.	737 (75.1)
6.Specific drugs are available to treat severe cases of COVID-19.	793 (80.8)
**Transmission Mode**	
7.COVID −19 can be spread through close contact with an infected person.	871 (88.8)
8.COVID −19 can be spread by droplets produced when you cough, sneeze or talk to an infected person.	895 (91.2)
9.Asymptomatic infected persons may become the source of the COVID − 19 infection.	786 (80.1)
**Preventive measures and treatment**	
10.Cover your nose and mouth completely with a tissue when coughing or sneezing or bend your elbow to prevent COVID − 19 transmission.	647 (66.0)
11.Saltwater gargling is effective in preventing COVID − 19 transmission.	697 (71.0)
12.Drinking strong alcohol can effectively prevent COVID − 19 transmission.	832 (84.8)
13.Wearing a mask can prevent COVID −19 transmission.	870 (88.7)
14.Influenza vaccination is an effective means of preventing COVID −19 transmission.	637 (64.9)
15.Avoiding crowded places can help prevent transmission of COVID − 19.	887 (90.4)
16.Traditional Chinese medicine can be used to treat COVID −19 cases.	616 (62.8)
**Mortality rate**	
17.So far, public data show that the mortality rate of the COVID −19 pneumonia is higher than MERS (Middle East Respiratory Syndrome).	409 (41.7)
18.So far, public data show that the mortality rate of the COVID −19 pneumonia is lower than that of SARS (Severe Acute Respiratory Syndrome).	539 (54.9)
**Average accuracy**	73.6

**Table 3:** Descriptive statistics of the explanatory variables related to COVID-19 (*N* = 981)

Items	Never n(%)	Rarely n(%)	Occasionally n(%)	Sometimes(%)	Frequently n(%)	Usually n(%)	Every time(%)
1.Print media use	254 (25.9)	184 (18.8)	158 (16.1)	137 (14.0)	113 (11.5)	95 (9.7)	40 (4.1)
2.Broadcast media use	47 (4.8)	82 (8.4)	167 (17.0)	245 (25.0)	244 (24.9)	144 (14.7)	52 (5.3)
3.Internet media use	6 (0.6)	5 (0.5)	8 (0.8)	40 (4.1)	105 (10.7)	283 (28.8)	534 (54.4)
4.Interpersonal communication	15 (1.5)	47 (4.8)	144 (14.7)	208 (21.2)	217 (22.1)	299 (30.5)	51 (5.2)
5.Public communication	35 (3.6)	144 (14.7)	211 (21.5)	292 (29.8)	192 (19.6)	89 (9.1)	18 (1.8)
6.Perceived salience of information	18 (1.8)	6 (0.6)	10 (1.0)	86 (8.8)	86 (8.8)	404 (41.2)	371 (37.8)
Average rate	6.4	8.0	11.9	17.1	16.3	22.3	18.1

### The existence of the knowledge gap of COVID-19

Analysis of variance revealed significant differences in knowledge levels in education levels (*P* < 0.01). Table 4 shows the variance analysis results of education level and other explanatory variables. Bivariate correlation analysis (Table 5) showed that there was a positive correlation between the education level and the knowledge level (R = 0.228, *P* < 0.01), indicating that there was a knowledge gap on COVID-19. So, hypothesis 1 was supported, suggesting that education level predicted the knowledge level gap.

**Table 4:** Results of variance analysis

	Education level (Mean ± SD)					F	p
	1.0(*n* = 71)	2.0(*n* = 81)	3.0(*n* = 150)	4.0(*n* = 490)	5.0(*n* = 189)		
Perceived salience of information	5.92 ± 1.42	5.81 ± 1.40	5.97 ± 1.23	6.21 ± 1.06	6.29 ± 0.85	4.614	0.001**
Interpersonal communication	4.57 ± 1.48	4.48 ± 1.56	4.93 ± 1.41	4.93 ± 1.24	4.87 ± 1.33	2.989	0.018*
Public communication	4.31 ± 1.52	4.56 ± 1.08	4.47 ± 1.34	4.22 ± 1.28	3.72 ± 1.21	10.187	0.000**
Broadcast media	4.89 ± 1.54	5.09 ± 1.16	4.84 ± 1.46	4.39 ± 1.44	3.85 ± 1.38	17.242	0.000**
Print media	3.70 ± 1.93	4.04 ± 1.68	3.79 ± 1.91	2.97 ± 1.74	2.36 ± 1.60	22.79	0.000**
Internet media	5.45 ± 1.57	5.96 ± 1.29	6.16 ± 1.11	6.40 ± 0.85	6.51 ± 0.76	19.693	0.000**

* *p* < 0.05 ** *p* < 0.01

**Table 5:** Bivariate correlation between independent and dependent variables

	knowledge							
Education	0.228**	Education						
Area	−0.045	−0.078*	Area					
Print media	−0.323**	−0.263**	− 0.028	Print media				
Broadcast media	−0.122**	−0.234**	0.004	0.542**	Broadcast media			
Internet media	0.362**	0.263**	0.028	−0.230**	0.064*	Internet media		
Interpersonal communication	−0.026	0.078*	−0.037	0.237**	0.214**	0.069*	Interpersonal communication	
Public communication	−0.149**	−0.153**	0	0.501**	0.539**	−0.019	0.365**	Public communication
Perceived salience of information	0.286**	0.125**	0.002	−0.095**	0.02	0.268**	0.166**	0.089**

* *p* < 0.05 ** *p* < 0.01

### Factors influencing the prediction of the knowledge gap

Table 5 shows that Internet media use and perceived salience of information also have a significantly positive correlation with the knowledge level in addition to the education level. On this basis, multi-layer regression analysis was carried out as the following: First, inputting the education level into group 1 as a control variable; Secondly, inputting Internet media use and perceived salience of information into group 2 as random variables, which were significantly positively correlated with the knowledge level; Finally, the interactive variables of education levels with Internet media use and perceived salience of information were input into group 3. The results are shown in Table 6, and both hypothesis 2 and hypothesis 3 were supported. Internet media use predicted the difference in knowledge level (B = 0.357, *P* < 0.01), and perceived salience of information (B = 0.233, *P* < 0.01) also significantly predicted the difference in knowledge level.

**Table 6:** Results of hierarchical regression analysis (*n* = 981)

	Model 1					Model 2					Model 3				
	B	SE	t	p	β	B	SE	t	p	β	B	SE	t	p	β
Constant	4.043**	0.144	28.172	0	–	0.804**	0.289	2.78	0.006	–	2.114**	0.757	2.792	0.005	–
Education level	0.275**	0.038	7.327	0	0.228	0.158**	0.036	4.377	0	0.131	−0.272	0.224	−1.218	0.224	−0.225
Internet media						0.357**	0.04	8.884	0	0.274	0.129	0.1	1.29	0.197	0.099
Perceived salience of information						0.233**	0.036	6.541	0	0.196	0.240*	0.105	2.285	0.023	0.203
education*internet media											0.075*	0.03	2.49	0.013	0.462
education*perceived salience of information											−0.004	0.03	−0.13	0.897	−0.024
R ^2^	0.052					0.185					0.191				
Adjusted R ^2^	0.051					0.183					0.187				
F	F (1,979) =53.689, *p* = 0.000					F (3,977) =74.096, *p* = 0.000					F (5,975) =45.964, *p* = 0.000				
△R ^2^	0.052					0.133					0.005				
△F	F (1,979) =53.689, p = 0.000					F (2,977) =79.970, p = 0.000					F (2,975) =3.253, *p* = 0.039				

Dependent variable: Score of the COVID-19 knowledge

* *p* < 0.05 ** *p* < 0.01

**Fig. 1:**
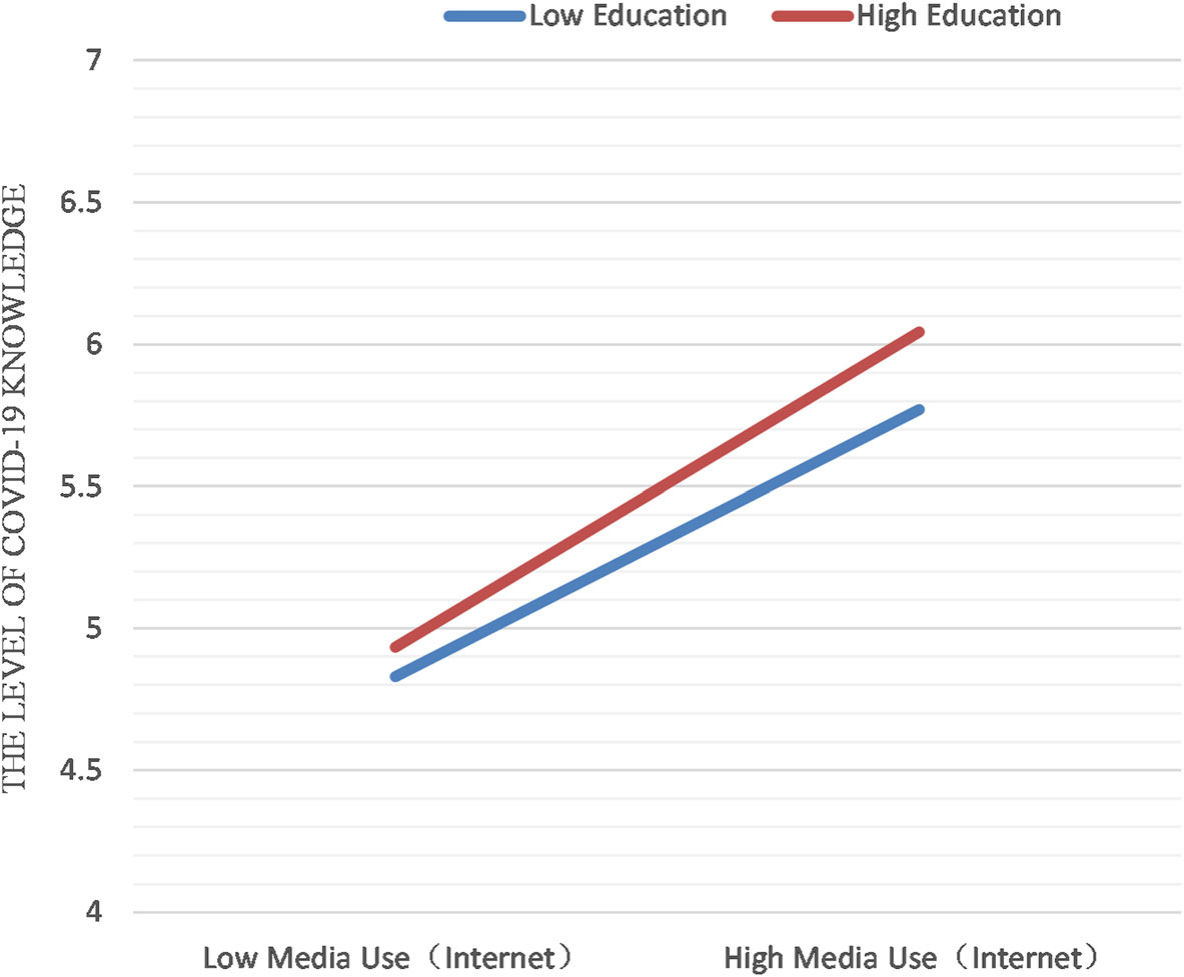
Regression plot for the interaction between media use (Internet) and education

In group 1, the education level significantly predicted the knowledge level of COVID-19 (*P* < 0.05), explaining 5.2% of the total variance of the outcome variable (β = 0.228, *P* < 0.01). After controlling for the education level, Internet media use and perceived salience of information in group 2 explained 13.3% of the total variance. Among them, Internet media use contributed the most (β = 0.274, *P* < 0.01). The regression analysis results with the introduction of interactive variables showed a significant interaction between the educational level and Internet media use. Internet media use could significantly predict the difference of knowledge level of COVID-19 caused by different educational levels, named as a knowledge gap. Figure 1 further shows the interaction model between the frequency of Internet media use and the education level. The increased frequency of Internet media use further expands the knowledge gap between high-educated and low-educated groups. In other words, the greater the use of Internet media, the wider the knowledge gap between higher-educated groups and lower-educated groups (β = 0.462, *p* < 0.05).

## Discussion

### COVID-19 knowledge gap and its formation mechanism

Academics have found the differences between the groups on the knowledge of science and put forward “science knowledge gap” [26] and “gap in scientific knowledge.” [27] This study in China found that the “COVID knowledge gap” exists in the public health science area of “COVID-19”. In traditional knowledge gap research, education is usually used as an operational indicator of socioeconomic status (SES) to explain the formation mechanism of a knowledge gap. The mechanism of education levels leading to the knowledge gap is that education has cultivated people’s ability of cognition and information processing, that is, the ability to learn new knowledge. Higher-educated people have an advantage in understanding complex information over lower-educated people [28]. This study found significant differences in the knowledge level of COVID-19 among groups with different educational levels, and higher-educated groups have a higher level of knowledge. As for COVID-19 is an unknown and a completely new knowledge field to both people with high and low education levels. The results reflected that the higher-educated groups have a better ability of information acquisition and information processing (knowledge learning), which leads to the difference in knowledge levels.

The media is an essential source for the public to learn different knowledge, including health knowledge. Various sources of information influence public perception of health issues and further influence people’s health behaviors. This study found that the predictive ability of Internet media was more significant than other sources of information related to COVID-19. Besides, traditional media, interpersonal communication, and public communication did not predict the increase of knowledge levels. It is also very consistent with the characteristics of people’s media use in the digital age. The Internet has become one of the important ways for people to obtain information. This study also suggested that Internet media was the most frequent and reliant way for people to get COVID-19 information (M = 6.28), and there was no regional difference. It also showed that the COVID-19 knowledge gap is essentially a digital divide concerning scientific knowledge. From the perspective of media information dissemination, there is a difference in the information dissemination efficiency of different media, and the improvement of the efficiency could improve health knowledge levels [29]. As the occurrence of the new virus, people have limited knowledge about COVID-19, and their perception of it is being updated. In terms of communication efficiency, the transmission speed, internal capacity, and update speed of COVID-19 related knowledge on Internet media are better than print media. Therefore, it can be explained that there is a difference in the prediction of the COVID-19 knowledge gap between the frequency of Internet media use and traditional media use. A study on the knowledge gap related to cancer confirmed that differences in preferred information content across media led to differences in the prediction of knowledge scores by frequency of media use [29]. In this study, the inability of radio, television, and print media to predict the rise in COVID-19 knowledge is also related to differences in media coverage. For example, we analyzed reports from China’s authoritative mainstream during February 2020 and found that the content of print media focused more on the development of the epidemic and the progress of anti-epidemic. The proportion of knowledge about COVID-19 in print media was relatively small.

Unlike the traditional media era, the formation of the knowledge gap in the digital era depends more on the active factors of individual information acquisition and processing. This study also found that perceived salience of information (usefulness and relevance) can significantly predict knowledge levels. It is consistent with previous studies’ findings that different groups’ perception of disease-related information leads to differences in knowledge scores [30].

Furthermore, studies revealed that individuals’ motivations, such as perceived information value and perceived content relevance, can affect their enthusiasm for media use [31]. This study also confirmed that perceived salience of information is positively correlated with Internet media use (*P* < 0.01). The variance analysis of this study showed that the perceived salience of information with different educational levels was significantly different (F = 4.614, *P* < 0.01), and the Internet media users with higher educational levels were more significant in perceived salience of information. Therefore, it can be inferred that the higher-educated groups have a firmer belief in the usefulness and value of knowledge and have a stronger motivation for information acquisition. They are usually active in following and learning knowledge and are more likely to improve their scientific knowledge through media use [32]. So, when investigating the influencing mechanism of COVID-19 knowledge level, a better explanatory framework for forming the knowledge needs both information sources and mediating factors. For example, personal motivations for information acquisition as mediating factors refer to the perceived value (interest and concern in an issue) and the initiative to acquire information. It also provides an entry point for further research.

### Internet media use and the expansion of the knowledge gap

As this study found, the COVID-19 Knowledge Gap is essentially a digital divide. The Digital Divide emphasizes that differences in the access and use of the Internet between different social groups lead to gaps in knowledge levels [33]. The gap in access to the Internet at the physical conditions for different groups is called the first digital divide, or access gaps [34]. In the era of digital globalization, the access gap among digital users is gradually being overcome. According to the 2021 China Internet Development Report released by the Internet Association of China, China had 989 million Internet users by the end of 2020, and the Internet penetration rate reached 70.4%. In particular, the total number of mobile Internet users exceeded 1.6 billion [35]. At the same time, this study found that Internet media is the most frequent and dependent way for people to obtain COVID-19 information. Therefore, access gaps are not necessarily the main barrier to COVID-19 knowledge levels.

However, having the same chance to be close to Internet media does not mean that people will use the Internet in the same way. The difference in digital skills and ability to use is considered the second digital divide, or use gaps, which refers to the difference in the time spent online, the purpose of using the Internet, and the online activities among different groups [36]. Access to online services anytime and anywhere does not mean the equivalence of information reception. The digital divide has spread from the first level of the “access gap” to the second level of the “use gap,” which is the difference in intensity, behavior, content, literacy, and other aspects when using the Internet media [37]. Regarding the influence of Internet media use, the lower socio-economic groups are not “increasingly impoverished” in absolute terms of knowledge. On the contrary, the knowledge level of all social classes has been correspondingly improved. However, the knowledge gap between the two groups widened due to the difference in the speed of improving knowledge levels. Many empirical studies showed that the new online communication technology had exacerbated the existing knowledge gap [38–40].

Studies have found that the digital divide is related to people’s habits and ability to use the Internet. People with high education are better at actively looking for information on the Internet, and they pay more attention to the informative and instrumental use of Internet media. In contrast, people with low education mostly regard the Internet as a tool for relaxation, and their usage habits mainly reflect their emphasis on the online entertainment function [38]. These usage habits may have influenced the way they acquired knowledge to some extent. In China, groups with high education levels tend to acquire scientific knowledge from knowledgeable and professional platforms such as Zhihu (China’s most popular Q&A platform, similar to Quora). In contrast, groups with low education levels tend to acquire knowledge and information from popular platforms such as Douyin (the Chinese version of TikTok) [41]. Different platforms make significant differences in knowledge presentation, depth, and professionalism. Besides, scientific information on the Internet presents unstructured, with relevant knowledge and information often interlinked through hyperlinks. For high-educated users, they tend to be better at handling unstructured information. Therefore, there is a significant gap in the use of Internet media among groups with different educational levels. Groups with higher educational levels likely acquire knowledge from the Internet, leading to a greater knowledge gap [42].

In addition, while providing massive information, Internet media also spread false and wrong information. During the COVID-19 crisis, much misinformation and rumors appeared on the Internet, becoming a “secondary disaster.” The difference in education level reflects the difference in media literacy from another side, which further influences the difference in knowledge levels. It can be seen that the availability of information does not always lead to great understanding [43]. Rather than Internet media use leading to the knowledge gap, it is the ability to use Internet media or Internet media literacy, such as filtering, evaluating, and discriminating information, that leads to the difference in the level of knowledge acquisition.

### How to bridge the knowledge gap and improve communication inequality?

In response to the COVID-19 knowledge gap, improving people’s knowledge level and fostering positive attitudes to pandemic preparedness is essential to enhance protective behavior against the pandemic. Thus, it is necessary to rethink the possibility and countermeasures of bridging the knowledge gap in the context of online communication.

#### Improving the pertinence in communication ways and contents

Although mass-media information campaigns cannot solve the information inequality caused by the difference in education levels, the communication of COVID-19 information in Internet media still provides channels and opportunities for low-educated groups to increase their knowledge. It also proves a widely accepted view in knowledge gap research, which is to increase publicity and the subsequent repetition of information to stimulate knowledge growth [44].

However, given the knowledge gap caused by the use gap, it is necessary to carry out targeted popular science communication according to the media use of different groups. The dissemination of scientific content should be more in line with the target audience’s knowledge level and information needs. On this occasion, even less-educated people can better grasp scientific knowledge because of the accessible way. It is necessary to fully understand the public and provide relevant information to attract their interest and increase their motivations to engage in health and science communication.

For example, in terms of information content, media can release more knowledge about the transmission and prevention of COVID-19 than the knowledge about the characteristics of the disease, which can directly promote public awareness of disease prevention. In the form of communication, media can innovate the dissemination of COVID-19 related knowledge, produce creative content and increase the perceived salience of COVID-19 knowledge among the audience. It is necessary to make a targeted communication to combat the COVID-19 pandemic according to people’s occupation, education level, and living residence [45]. Also, big data technology can analyze users’ preferences and behaviors and design content based on the user behavior data [46].

#### Building authoritative scientific knowledge communication platforms

Theoretically, multi-channel, unlimited, interactive, and multimedia scientific information on the Internet does provide a vast range of scientific content and knowledge for people of all socioeconomic status. According to the above findings, Internet media use challenges lower-educated groups rather than bridges the scientific knowledge gap. In 2013, *Science* pointed out that the scientific information sources online may narrow the knowledge gap [47]. Building COVID-19 knowledge popularization platforms with high credibility, such as mainstream media, WeChat official accounts, and Zhihu, makes it easier for the public to access various controversial sciences. The public can improve their knowledge levels and make up for the lack of formal education in understanding scientific knowledge via the platforms.

#### Developing internet media literacy and scientific literacy

Science is essential to understanding the world, society, the environment, and even the epidemic. Scientific knowledge is of increasing importance to the daily life of the public. It is especially reflected in the infodemic that spread along with COVID-19 in 2020. For example, the false information of “drinking Shuanghuanglian oral liquid and highly alcoholic liquor can prevent and resist COVID-19” spread widely in society. Misinformation increased the “information entropy” of the whole Internet field and brought a severe challenge to the public’s cognition and rationality.

In this context, compared with the traditional media era, which relies on the filtering and purification of information by professional gatekeepers, the public needs to rely more on their rationality nowadays. With high media and scientific literacy, the public can identify pseudoscience and rumors, rid fear, establish autonomy and replace fantasy with knowledge. Thus, strong public communication education can improve public media and scientific literacy and narrow the “digital divide” caused by differences in technology use and decoding capabilities.

In the face of COVID-19, how to improve the effectiveness of science communication and enhance health knowledge and awareness has great practical significance. The findings of this study are helpful for health promotion, such as promoting vaccination. In terms of media use, it is a breakthrough to use Internet media platforms, such as Douyin and Kuaishou, to promote vaccination in an easy-to-understand way. From the perceived significance of information, the benefits of vaccination can be emphasized in terms of its relevance and usefulness to everyone.

### Implications and limitations

This study is based on the communication situation and media environment of COVID-19 in China. Although there are many studies on the knowledge gap between mass media and health issues, there are still few specific studies on the knowledge gap related to COVID-19. This study attempts to fill the gap in such research. In addition, with the further development of new media and information technology, the research on the unequal distribution of health information brought by digital technology is of great practical value.

Also, there are several limitations to this study. First of all, although the volunteer samples in this study involved 30 provinces (autonomous regions, cities), the sample composition is uneven, which mainly are urban residents, young people, and people with a college education or above. These groups have the advantages of using online media, limiting exploring the frequency of media use in this study. Future studies can further expand the sample size or investigate other areas. Secondly, the knowledge gap between people of different socio-economic status (SES) is usually a long-term rather than a short-term phenomenon, and knowledge is not immutable [44]. The cross-sectional study cannot fully reflect this phenomenon, and a longitudinal study can be attempted in the future. Finally, the factors affecting knowledge about COVID-19 listed in this study are not comprehensive. Future research can further explore different aspects such as motivation, differences in media literacy to make up for this deficiency.

## Conclusions

Although the media environment has changed from traditional to social media, the “knowledge gap” has always existed. This study further proved the existence of the digital knowledge gap of COVID-19. In this study, education levels can significantly predict the knowledge gap of COVID-19. The perceived salience of information can significantly predict the difference in the knowledge level of COVID-19 but cannot change the knowledge gap caused by the difference in education levels. Only Internet media use and education level have an interactive effect on forming the COVID-19 knowledge gap. That is, Internet media use will expand the COVID-19 knowledge gap between groups with different education levels. However, traditional media such as newspapers, radio and television, public communication, and interpersonal communication did not improve the knowledge level. These findings help us understand the knowledge gap and its formation mechanism, providing a reference for promoting health communication.

## Data Availability

The datasets used and analyzed during the current study available from the corresponding author on reasonable request.

## Abbreviations

COVID-19: coronavirus disease
WHO: World Health Organization
SES: Socioeconomic status
